# Hybrid Dietary-Blood Inflammatory Profiles and Postmenopausal Breast Cancer: A Case-Control Study

**DOI:** 10.3390/nu12113503

**Published:** 2020-11-14

**Authors:** Beata Stasiewicz, Lidia Wadolowska, Maciej Biernacki, Malgorzata Anna Slowinska, Marek Drozdowski

**Affiliations:** 1 Department of Human Nutrition, Faculty of Food Sciences, University of Warmia and Mazury in Olsztyn, Sloneczna 45f, 10-718 Olsztyn, Poland; lidia.wadolowska@uwm.edu.pl (L.W.); malgorzata.slowinska@uwm.edu.pl (M.A.S.); 2 Department of Surgery, University of Warmia and Mazury in Olsztyn, 11-041 Olsztyn, Poland; maciej.biernacki@uwm.edu.pl; 3 Department of Psychology and Sociology of Health and Public Health, School of Public Health, University of Warmia and Mazury in Olsztyn, 11-041 Olsztyn, Poland; marek.drozdowski@uwm.edu.pl

**Keywords:** breast cancer, food, diet, inflammation, C-reactive protein, Interleukin-6, leukocyte

## Abstract

The carcinogenesis process is associated with inflammation, which can be modified by diet. There is limited evidence regarding the inflammatory status and diet in association with breast cancer (BC). The aim of this study was to investigate the association of hybrid dietary-blood inflammatory profiles (HD-BIPs) with postmenopausal breast cancer occurrence. The case-control study was conducted among 420 women (230 controls, 190 primary BC cases) aged 40–79 years from north-eastern Poland. Blood levels of C-reactive protein (CRP), interleukin-6 (IL-6) and leukocyte count were marked in 129 postmenopausal women (82 controls, 47 cases). The 62-item food frequency questionnaire (FFQ-6) was used to the dietary data collection. Two HD-BIPs were found using the Principal Component Analysis (PCA). The “Pro-healthy/Neutral-inflammatory” profile was characterized by the frequent consumption of wholemeal cereals/coarse groats, legumes, vegetables, fruits, nuts/seeds and fish. The “Unhealthy/Pro-inflammatory” profile was characterized by the frequent consumption of red/processed meats, animal fats, sugar/honey/sweets, refined cereals/fine groats, and an increased concentration of CRP, IL-6 and granulocyte-to-lymphocyte ratio. The lower odds ratio (OR) of breast cancer was associated with the higher adherence to the “Pro-healthy/Neutral-inflammatory” profile (OR = 0.38; 95% Cl: 0.18–0.80; *p* < 0.01 for the higher level vs. lower level, crude model; OR for one-point score increment: 0.61; 95% Cl: 0.42–0.87; *p* < 0.01, adjusted model). The higher OR of breast cancer was associated with the higher adherence to the “Unhealthy/Pro-inflammatory” profile (OR = 3.07; 95%Cl: 1.27–7.44; *p* < 0.05 for the higher level v.s. lower level, adjusted model; OR for one-point score increment: 1.18; 95%Cl: 1.02–1.36; *p* < 0.05, adjusted model). This study revealed that the consumption of highly processed, high in sugar and animal fat foods should be avoided because this unhealthy diet was positively associated with postmenopausal breast cancer occurrence through its pro-inflammatory potential. Instead, the frequent consumption of low-processed plant foods and fish should be recommended since this pro-healthy diet was inversely associated with the cancer occurrence even though its anti-inflammatory potential has not been confirmed in this study sample.

## 1. Introduction

Breast cancer (BC) is the most commonly diagnosed cancer and the leading cause of cancer deaths in women worldwide [1]. Based on the latest global cancer statistics from 2018, there were 2.1 million new cases of BC and 627,000 deaths of this cause worldwide, which accounted for 24.2% of all female cancer cases and 15.0% of all female cancer deaths [2]. Breast cancer is one of the main problems for women’s health and its occurrence has been growing in many countries, both developed and developing [1]. In Poland, breast cancer is the most frequently diagnosed cancer and the second cause of death from cancer in women [3]. According to the latest data from 2013, the number of BC cases was 17,142, including 5816 deaths, which accounted for 21.9% and 13.9% of all female cancer cases and deaths in Polish women, respectively [3]. The incidence of BC increases with age. It was estimated that approximately 80% of BC incidence is noted in postmenopausal women, with half of all BC cases being diagnosed between 50–69 aged [3].

Breast cancer aetiology is complex and besides the age and genetic well-known risk factors, there is increasing evidence that inflammation is associated with the cancer development [4,5,6]. Chronic inflammation within the breast tissue can lead to the different stages of carcinogenesis, including cancer initiation through the breast epithelial cell transformation and apoptosis inhibition, promoting proliferation, stimulating angiogenesis, migration and metastasis [5,6]. A possible mechanism by which inflammation can contribute to carcinogenesis includes inflammation-induced oxidative stress, which is the source of pro-inflammatory cytokines and reactive oxygen species [6]. In turn, these pro-inflammatory mediators may cause DNA damage and genomic instability, which deepens inflammation within the tissue [5,6].

There are a variety of inflammatory biomarkers, which may be involved in carcinogenesis [7,8,9,10,11,12,13,14,15,16,17,18,19,20]. One of the major pro-inflammatory cytokines produced by cells in inflamed tissue is Interleukin-6 (IL-6) [7,8,9,10]. IL-6 has an important role in chronic inflammatory diseases and may play a role in cancer development [7,8,9,10]. However, the current evidence of the link between the IL-6 and BC risk is insufficient [7,8]. The next, widely used marker of systemic inflammation is non-specific C-reactive protein (CRP), hepatic-synthesized in response to pro-inflammatory cytokines [7,8,9,10,11,12,13,14,15,16,17,18,19,20]. CRP has been recognised as a risk factor for BC in epidemiologic studies [18,19]. However, results from prospective studies remain inconsistent [12,13,14,15]. Blood leukocyte count (white blood count; WBC) is another routinely used inflammatory marker related to the immune system’s response [18,19,20]. Elevated WBC concentration has been associated with an increased risk of several cancers, including breast cancer [18]. However, the role of WBC components in the risk of BC has not yet been sufficiently investigated. Recently, neutrophil-to-lymphocyte (N/L) and granulocyte-to-lymphocyte (G/L) ratios were found to be predictors of mortality in breast cancer patients [20], although there is a lack of data on their associations with BC risk.

Inflammation could be affected by many modifiable lifestyle factors, including smoking, alcohol drinking, physical activity and dietary factors, through the modulation of inflammatory cytokines [4,6]. There is growing evidence that some food items and specific dietary components, such as red meat, saturated fatty acids, refined carbohydrates, and a high omega-6 to omega-3 fatty acid ratio, may have a pro-inflammatory potential, whereas some others, including fruits, vegetables, fish, whole grains, legumes and flavonoids, may have an anti-inflammatory potential [21,22,23,24,25,26,27]. The literature-based, quantitative dietary inflammatory index (DII) for pro- and anti-inflammatory dietary factors [28,29,30] is widely used to evaluate the inflammatory potential of diet in relation to inflammatory diseases. Although the results of some studies suggest a positive association between DII and the risk of cancer [28,29,31,32], the role of diet in the modulation of inflammation in the aetiology of breast cancer remains unclear [33,34,35].

Despite the increasing interest in the inflammatory potential of the influence of diet on breast cancer incidence [28,29,30,31,32,33,34,35], the available studies were focused on examining the association in one of three directions, showing a link between a diet with inflammation [21,22,23,24,25,26,27], or diet with breast cancer [36,37,38,39] or a single inflammatory biomarker with breast cancer [7,8,9,10,11,12,13,14,15,16,17,18,19,20]. Due to the complex interactions between diet and inflammation [21], as well as the role of inflammation in cancer pathogenesis [6], it is important to study the combined association of dietary and inflammatory factors, expressed in the usual food consumption and a broader panel of inflammatory biomarkers in relation to the breast cancer. 

The aim of this study was to investigate the association of hybrid dietary-blood inflammatory profiles (HD-BIPs) with postmenopausal breast cancer occurrence. In evaluating this association, many potential confounders, including molecular subtypes of cancer, lifestyle and reproductive factors were considered.

## 2. Materials and Methods 

### 2.1. Ethical Statement

The present study was approved by the Bioethics Committee of the Faculty of Medical Sciences, University of Warmia and Mazury in Olsztyn on 2 October 2013 (Resolution no. 29/2013) [40]. All subjects gave their written and informed consent to participate in the study.

### 2.2. Study Design and Sample Collection

The total case-control sample obtained 420 women, aged 40.0–79.9 (mean 59.9) years. The general inclusion criteria were: (i) women, (ii) age ≥18 years, (iii) north-eastern Poland (urban and rural areas), (iv) breast ultrasonography and/or mammography, and (v) consent to participate in the study. Pregnant women were excluded from the study. 

The inclusion criterion of the cancer sample collection was a primary diagnosis of breast cancer (from 7 to 28 days before recruitment in the study) confirmed in medical records (biopsy and histopathology), before any treatment or surgery. All breast cancer cases were patients of the Warmia and Mazury Cancer Centre of the Ministry of the Interior and Administration’s Hospital. Regarding the hormone receptor status of breast cancer, the most frequently diagnosed (74%) were Luminal A tumours with positive receptor status of oestrogen (ER+) and/or progesterone (PR+), and negative human epidermal growth factor receptor 2 (HER2-). The inclusion criterion of the control sample collection was a lack of any breast pathology confirmed in the ultrasonography and/or mammography screening made within six months before recruitment to the study.

**Figure 1:**
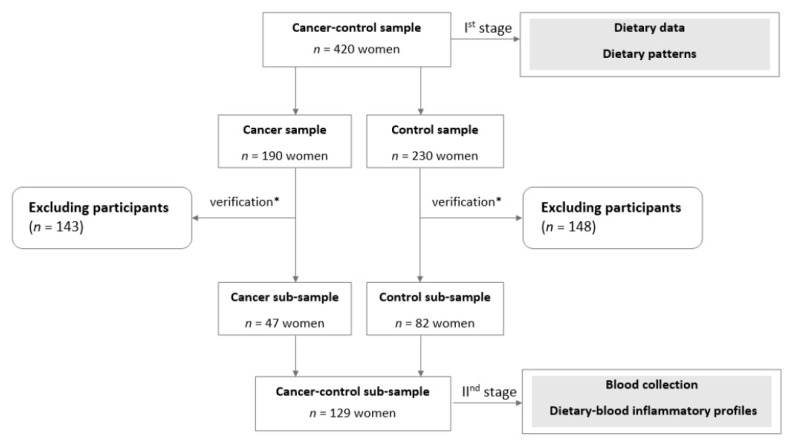
Study design and sample collection; verification* after including additional inclusion and exclusion criteria (details were described in Section 2.2).

Besides the abovementioned basic sample collection criteria, in the next stage of the study (II nd stage, Figure 1), to avoid the possible impact of many factors on the outcomes of blood markers, the additional, more restrictive inclusion criteria of cancer-control sub-sample collection were considered: (i) post-menopausal status (natural menopause), (ii) consent to blood collection, (iii) fasting status at the time of blood sampling, and (iv) lack of chronic diseases or infections and no use of medication (particularly anti-inflammatory). Therefore, women after a hysterectomy with a non-fasting status and those diagnosed with diabetes, atherosclerosis, infections, autoimmune diseases or hormonal disorders were excluded from the study. Finally, in the present study, the cancer-control sub-sample obtained 129 postmenopausal women, aged 45.0–79.9 (mean 61.9 SD 8.2) years, including 82 controls (control sub-sample) and 47 BC cases (cancer sub-sample; Figure 1). Detailed characteristics of the cancer-control sample and its sub-sample are shown in Supplementary Tables S1 and S2. 

### 2.3. Dietary Data

Dietary data were obtained for 420 women (190 BC cases, 230 controls) [40]. All subjects provided information about the frequency of food consumption at least 12 months prior to being recruited into the study. The 62-item food frequency questionnaire (FFQ-6) in an interviewer-administered version was used [41]. The consumption frequency was recalculated in times/day according to the scheme given below Table 1. Next, the consumption frequency of some food items were aggregated from ten food groups: sugar/honey/sweets, wholemeal cereals/coarse groats, refined cereals/fine groats, animal fats, fruits, vegetables, nuts/seeds, legumes, red/processed meats and fish (Table S3). This selection was made based on the literature-derived pro- or anti-inflammatory potential of these food groups [28]. These data were included in the further analysis performed for 129 postmenopausal women (47 BC cases, 82 controls). Alcohol or sweetened beverages and energy drinks were not included in the current study due to their occasional consumption. Alcohol drinking, as a well-established risk factor for breast cancer [4], was included in the set of confounders. 

### 2.4. Blood Markers

Blood samples were obtained for 129 postmenopausal women (47 BC cases, 82 controls). Details of the laboratory procedures regarding the blood samples collection and serum obtainment were described previously [40,42]. C-reactive protein (CRP) concentrations were marked in serum samples using a Cobas Integra 400 plus auto-analyzer (Roche Diagnostics^®^, Basel, Switzerland). Serum concentrations of Interleukin-6 (IL-6) were marked with electrochemiluminescence immunoassays (ECLIA) using an automated immune-analyser Cobas e411 (Roche Diagnostics^®^, Basel, Switzerland). The blood concentration of haematological parameters with the division of leukocytes into granulocytes, including neutrophils and agranulocytes (including lymphocytes), were measured using a haematological analyzer MEK-7300 (Nihon Kohden^®^, Tokyo, Japan). The counts of leukocytes and their classes were measured using a detecting electrical resistance method, and light scattering (flow cytometry using a semiconductor laser [43]), respectively. The measurement ranges were as follows: CRP 1.0–200mg/L, IL-6 1.5–5000pg/mL, and leukocytes, including neutrophils and lymphocytes 0–299 × 10^3^ cells/µL. 

### 2.5. Hybrid Dietary-Blood Inflammatory Profiles

The hybrid dietary-blood inflammatory profiles (HD-BIPs) were derived using a Principal Component Analysis (PCA) with varimax rotation [44]. The input variables were the consumption frequency of ten food groups described above and the blood concentrations of three inflammatory markers (C-reactive protein, Interleukin-6 and granulocyte-to-lymphocyte (G/L) ratio). All continuous data were standardised before PCA to avoid the effect of the different dimensions. This allowed the standardised normal distributions N (mean 0, SD 1) of these variables to be obtained so that all sample data ranged between 0 and 1 [44]. Standardisation was performed automatically using the “Standardise” function of STATISTICA software (version 13.0 PL; StatSoft Inc., Tulsa, OK, USA; StatSoft, Krakow, Poland). The criteria which were considered in the PCA-derived HD-BIPs identification were described elsewhere [40,44]. HD-BIPs were labelled based on the main characteristic components with factor loadings ≥|0.30|, with the higher values indicating a stronger correlation between food consumption and inflammatory markers and a given profile. For each of the HD-BIPs, the scores (in points) as a sum of the product of food consumption frequency (times/day) and inflammatory markers values (blood concentration) and its factor loadings were calculated. Next, for each profile, two levels, based on the HD-BIPs score’s median, were created: (lower < Me, higher ≥ Me). The higher score or level indicated the higher adherence to the given profile, accordingly.

### 2.6. Statistical Analysis

The continuous variables (e.g., food consumption frequency expressed in times/day, blood concentration of single inflammatory biomarkers, scores of hybrid dietary-blood inflammatory profiles expressed in points) were showed as means and 95% confidence intervals (95% CI). The medians (Me) were used to transform continuous variables into the categorical variables (e.g., food consumption frequency expressed in categories, blood levels of single inflammatory biomarkers, levels of HD-BIPs), which were presented in percentages of the sample. Differences between groups were verified with Pearson’s chi-squared test (categorical data), Student’s t-test for continuous variables with a normal distribution (log-transformed and blood concentration of single inflammatory biomarkers) or Kruskal–Wallis test for continuous data without normal distribution (e.g., food consumption frequency expressed in times/day) [44].

The breast cancer occurrence in associations with the adherence to the HD-BIPs or blood levels of single inflammatory biomarkers was assessed using the logistic regression analysis. The odds ratios (ORs) and 95% CI were calculated. The references (OR = 1.00) were the control sample and the lower level of each HD-BIPs or a lower level of a single biomarker (ref.: < Me). The ORs of breast cancer for a 1-point increase in each HD-BIPs score and blood concentration of single inflammatory biomarkers were also calculated. Three models were created: crude model 1, model 2 adjusted for the confounders, and model 3 adjusted for the same confounders included in model 2 and also for dietary patterns (DPs) scores (for single inflammatory biomarkers only). The set of confounders included: age (years), BMI (kg/m^2^), socioeconomic status (low, average, high; Table S4), overall physical activity (low, moderate, high; Table S5), smoking status (non-smoker, smoker), alcohol drinking (times/day), age at menarche (<12, 12–14.9, ≥15 years), number of full-term pregnancies (0, 1–2, ≥3), oral contraceptive use (no, yes), hormone-replacement therapy use (no, yes), family history of breast cancer in first- or second-degree relative (no, I don’t know, yes), vitamin/mineral supplement use (no, yes) and molecular subtypes of breast cancer (triple-negative, ER-, PR-, HER2+ subtype, luminal A, luminal B). The significance level of OR was verified with Wald’s test [44]. The level of statistical significance was considered at *p* < 0.05. All statistical analyses were performed using STATISTICA software (version 13.0 PL; StatSoft Inc., Tulsa, USA; StatSoft, Krakow, Poland).

## 3. Results

### 3.1. Hybrid Dietary-Blood Inflammatory Profiles

**Figure 2:**
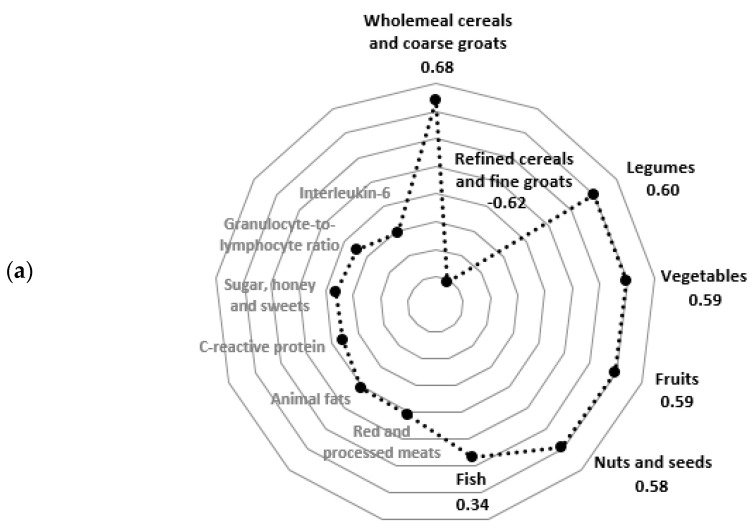

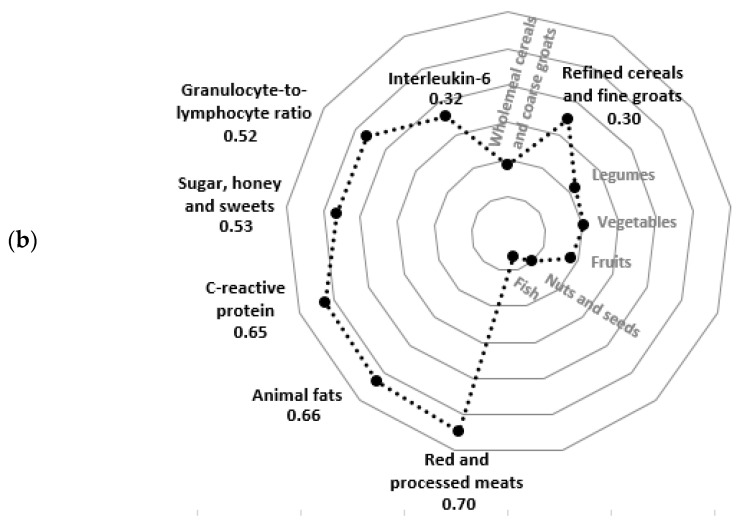
Diagrams of factor loadings for the frequency of food consumption and blood concentrations of inflammatory biomarkers in PCA-derived hybrid dietary-blood inflammatory profiles among postmenopausal women (*n* = 129): (**a**) “Pro-healthy/Neutral-inflammatory” profile; (**b**) “Unhealthy/Pro-inflammatory” profile. The main profile components with absolute factor loadings ≥ |0.30| have been bolded.

Two PCA-derived hybrid dietary-blood inflammatory profiles were identified (Figure 2). The total variance explained by two HD-BIPs was 35%. The “Pro-healthy/Neutral-inflammatory” profile was positively loaded by the consumption frequency of: wholemeal cereals and coarse groats (*r* = 0.68), legumes (*r* = 0.60), vegetables (*r* = 0.59), fruits (*r* = 0.59), nuts and seeds (*r* = 0.58) and fish (*r* = 0.34) and was negatively loaded by the consumption frequency of refined cereals and fine groats (*r* = –0.62). The “Unhealthy/Pro-inflammatory” profile was positively loaded by the consumption frequency of: red and processed meats (*r* = 0.70), animal fats (*r* = 0.66), sugar, honey and sweets (*r* = 0.53) and refined cereals and fine groats (*r* = 0.30) as well as by the blood concentration of: C-reactive protein (*r* = 0.65), granulocyte-to-lymphocyte ratio (*r* = 0.52) and Interleukin-6 (*r* = 0.32; Figure 2). Factor loading values for all profile components are shown in Supplementary Table S6.

The frequency of food consumption and blood concentration of single inflammatory biomarkers by levels of hybrid dietary-blood inflammatory profiles are shown in Table 1. The higher level of “Pro-healthy/Neutral-inflammatory” profile compared to the lower level of this profile was characterised by the higher mean consumption frequency of wholemeal cereals/coarse groats, fruits and vegetables (at least once a day), nuts/seeds, legumes and fish (at least several times/month; Table 1). The higher level of “Unhealthy/Pro-inflammatory” profile compared to the lower level of this profile was characterised by the higher mean consumption frequency of sugar/honey/sweets, animal fats and red/processed meats (at least once a day) and the higher consumption frequency of refined cereals/fine groats (at least several times/week). In the higher level of the “Unhealthy/Pro-inflammatory” profile, more than half of the women had an elevated level of CRP, leukocyte, granulocyte and neutrophil counts, as well as an elevated granulocyte-to-lymphocyte ratio (Table 1).

### 3.2. Hybrid Dietary-Blood Inflammatory Profiles and Breast Cancer

Breast cancer cases had a lower mean score in the “Pro-healthy/Neutral-inflammatory” profile than controls (0.09 versus 1.42 points; Table 2). The number of BC cases was lower among subjects representing the higher level of the “Pro-healthy/Neutral-inflammatory” profile than among the control subjects (35.4 versus 59.3%). On the other hand, BC cases had a higher mean score of the “Unhealthy/Pro-inflammatory” profile (6.73 versus 4.19 points) and more cancer cases had a higher level of this profile than controls (68.8 versus 39.5%). The sub-sample characteristics by levels of hybrid dietary-blood inflammatory profiles are shown in Supplementary Table S7. 

Compared to the controls, BC cases had a higher mean blood concentration of most of the analysed inflammatory biomarkers, i.e., interleukin-6 (4.06 versus 2.46 pg/mL), leukocyte count (7.10 versus 5.74 × 10^3^ cells/µL), including absolute granulocyte count (4.51 versus 3.26 10^3^ cells/µL), which were mostly neutrophils (4.39 versus 3.02 × 10^3^ cells/µL), as well as the mean ratio of granulocyte-to-lymphocyte (2.36 versus 1.70; Table 2). The histograms of interleukin-6 and leukocyte concentrations among cancer and control sub-samples are shown in Supplementary Figure S1. Compared to the controls, more cases of BC had elevated levels of blood concentration of leukocytes ≥5.95 × 10^3^ cells/µL (66.0 versus 40.7%), absolute granulocytes ≥3.56 × 10^3^ cells/µL (77.6 versus 33.3%), including neutrophils ≥ 3.31 × 10^3^ cells/µL (78.0 versus 33.3%), and the ratio of granulocyte-to-lymphocyte ≥ 1.75 (73.5 versus. 35.8%; Table 2). There were no significant differences in the blood concentration of C-reactive protein or agranulocytes (including lymphocytes) between cases and controls.

**Figure 3:**
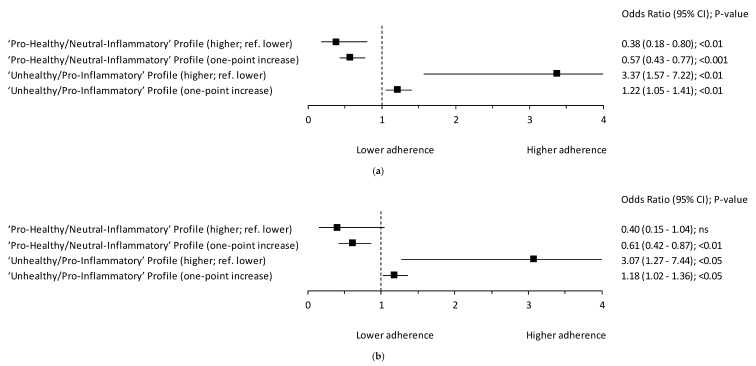
Forest plots of the association between the adherence to the PCA-derived hybrid dietary-blood inflammatory profiles among women (*n* = 129) and postmenopausal breast cancer occurrence: (**a**) crude model 1; (**b**) model 2 adjusted for: age (years), BMI (kg/m^2^), socioeconomic status (low, average, high), overall physical activity (low, moderate, high), smoking status (non-smoker, smoker), alcohol drinking (times/day), age at menarche (<12, 12–14.9, ≥15 years), number of full-term pregnancies (0, 1–2, ≥3), oral contraceptive use (no, yes), hormone-replacement therapy use (no, yes), family history of breast cancer in first- or second-degree relative (no, I don’t know, yes), vitamin/mineral supplement use (no, yes) and molecular subtypes of breast cancer (triple-negative, ER-, PR-, HER2+ subtype, luminal A, luminal B). ref.—referent, the reference categories were the control sample and the lower level of hybrid dietary-blood inflammatory profiles; CI—confidence interval; *p*-value—the level of statistical significance verified with Wald’s test; ns—statistically insignificant.

The odds ratio of breast cancer occurrence was lower in the higher level of the “Pro-healthy/Neutral-inflammatory” profile by 62% (OR = 0.38; 95% Cl: 0.18–0.80; *p* < 0.01; crude model 1; reference: lower level), however this association disappeared after adjustment for the set of confounders (adjusted model 2; Figure 3). A one-point increase in the “Pro-healthy/Neutral-inflammatory” profile decreased the occurrence of breast cancer by 39% (OR = 0.61; 95% Cl: 0.42–0.87; *p* < 0.01; adjusted model 2) and 43% (OR = 0.57; 95% Cl: 0.43–0.77; *p* < 0.001; crude model 1). The odds ratio of breast cancer occurrence was approximately three-times higher in the higher level of the “Unhealthy/Pro-inflammatory” profile (OR = 3.07; 95% Cl: 1.27–7.44; *p* < 0.05; adjusted model; reference: lower level, OR = 3.37; 95% Cl: 1.57–7.22; *p* < 0.01; crude model 1; reference: lower level; Figure 3). A one-point increase in the “Unhealthy/Pro-Inflammatory” profile increased the occurrence of breast cancer by 18% (OR = 1.18; 95% Cl: 1.02–1.36; *p* < 0.05; adjusted model 2) and 22% (OR = 1.22; 95% Cl: 1.05–1.41; *p* < 0.01; crude model 1). The results of the logistic regression analysis of the breast cancer occurrence by adherence to the blood concentration of single inflammatory biomarkers are shown in Supplementary Table S8. 

## 4. Discussion

This study presents the comprehensive approach in assessing the association of diet and inflammation as data-driven hybrid dietary-blood inflammatory profiles with postmenopausal breast cancer. These findings highlight the strong harmful association of the “Unhealthy/Pro-inflammatory” profile with the breast cancer occurrence, independent of many potential confounders, among women from north-eastern Poland. However, the data also revealed the beneficial association between the “Pro-healthy/Neutral-inflammatory” profile and breast cancer. 

### 4.1. ”Unhealthy/Pro-inflammatory” Profile and Breast Cancer

The findings provide interesting insights into the association of the dietary and inflammatory factors in breast cancer aetiology. A higher adherence to the “Unhealthy/Pro-inflammatory” profile was associated with greater than three-fold higher odds of postmenopausal BC occurrence among Polish women, and this strong association remained even after adjustment for many potential confounders. The “Unhealthy/Pro-inflammatory” profile was characterised by a relatively high frequent consumption of red and processed meats, animal fats, sugar/honey/sweets, refined cereals/fine groats, as well as by the relatively high blood concentration of CRP, IL-6 and the G/L ratio. Thus, these results indicated the possible interactions between the consumption of some foods and the inflammatory markers in association with BC. No previous studies contain data-driven profiles which included both dietary and inflammatory factors like those in the current study, so a clear comparison is not easy. In the available studies, a widely used tool to quantify the inflammatory potential of diet, related to cancer, is the hypothesis-driven dietary inflammatory index (DII), which also includes foods as components of the “Unhealthy/Pro-inflammatory” profile [28,29,30]. In line with the current findings, a more pro-inflammatory diet expressed by the higher DII increased the odds of BC by 4% and 14% in recent systematic reviews and meta-analyses [45,46], by 22% and 75% in a Swedish prospective study and an Italian case-control study [28,29], and by greater than two, and even three-fold in Chinese and Korean case-control studies, respectively [31,32]. Contrary to the present results, in a Women’s Health Initiative (WHI) cohort study, an Iowa Women’s Health study (IWHS) and a German case-control study [33,34,47], no significant associations between DII and overall breast cancer incidence were observed. These discrepancies in findings may be due to the differences in study design, sample size, data extraction or the number of food parameters-to-DII calculated [35,48]. No study has reported an inverse association of a pro-inflammatory diet with BC.

Findings from the current study strengthen previous studies showing that high adherence to the “Non-Healthy” dietary pattern (DP) increased the odds of breast cancer occurrence by approximately three times [40]. Both the “Non-Healthy” DP derived in the prior study, and the “Unhealthy/Pro-inflammatory” profile derived in the present study were positively loaded by the relatively high consumption frequency of red and processed meats, animal fats, sugar/honey/sweets and refined cereals/fine groats. Compared to previously-derived “Non-Healthy” DP, the “Unhealthy/Pro-inflammatory” profile was additionally positively loaded by the blood concentration of CRP and IL-6, as well as the G/L ratio. Thus, these results constitute further evidence that some of the food consumption may be pro-inflammatory, as measured by the levels of inflammatory markers [21]. Similar to the “Unhealthy/Pro-inflammatory” profile, the DII has been positively associated with several inflammatory markers, including CRP and Il-6 [28,29]. In regards to the single food items, Azadbakht et al. [27] reported a positive association between red meat consumption and serum levels of CRP. The potential mechanisms of the link between diet and inflammation are not well understood. However, there are some possible explanations for this association. Unhealthy dietary patterns characterised by the frequent consumption of highly processed foods and red meat with a high content of total fats and trans fatty acids increase the level of the free oxygen radicals, which generate oxidative stress and cause an increase in inflammation [21]. Moreover, refined foods with a high content of sugar increases systemic inflammation through increased insulin levels and stimulation of insulin-like growth factor receptors [6,21]. 

### 4.2. Inflammatory Markers and Breast Cancer

The positive association of the “Unhealthy/Pro-inflammatory” profile with breast cancer must also consider its inflammatory features. Unfortunately, the few available studies have focused mainly on the single inflammatory markers in association with BC. The “Unhealthy/Pro-inflammatory” profile was positively loaded by the serum concentration of CRP, which has been positively associated with postmenopausal breast cancer in a prospective nested case-control study within the EPIC-Varese cohort study [8] and several meta-analyses [11,12,13]. Results from the molecular studies suggest that CRP may be involved in breast cancerogenesis through the increased transcription of genes involved in inflammation and the interaction between inflammation and the estrogen pathway [49,50]. Nevertheless, no statistically significant association with BC occurrence was found when the single serum concentration of CRP was considered separately. The lack of significant differences in the CRP levels between BC cases and controls was also reported in Swedish, French and American cohort studies [7,12,15] and meta-analyses [11]. On the contrary, in a WHI study [19], and the Glasgow Inflammation Outcome Study [17], the CRP concentration was higher in the BC cases than in controls. This discrepancy could result from the use of different cut-off points of CRP and the time of CRP measurements (pre- or post-diagnosis) [11,12,13]. 

For another inflammatory component of the “Unhealthy/Pro-inflammatory” profile, a higher mean serum concentration of IL-6 was observed in BC cases compared to the controls among Polish women. Considering the single inflammatory markers, it was found that a one-unit increase (pg/mL) in serum IL-6 increased the odds of the BC occurrence by 50%. This result is in agreement with the findings of the EPIC-Varese cohort study, where a one standard deviation increase in plasma IL-6 (pg/mL) was associated with an increased risk of BC by 58% [8]. This positive association may be explained through the involvement of IL-6 in breast cancer cell migration and invasion by the activation of many factors, including estrogen [49]. However, some studies report no association between IL-6 and breast cancer, including the WHI study and the British Women’s Heart and Health Study [7].

The last inflammatory component of the “Unhealthy/Pro-inflammatory” profile was the ratio of granulocytes-to-lymphocytes. The G/L represents the major classes of leukocytes. Similar to the current study, in the WHI study, the mean leukocyte count was higher in BC cases than in controls (7.10 vs. 5.74, and 7.52 vs. 6.15 thousand cells/µL, respectively) [19]. These results support the role of leukocytes in the association between immune mechanisms, inflammation and BC development [20]. In line with the current study, where the mean G/L ratio was higher in BC cases than in controls (2.36 vs. 1.70), in a study conducted by Wulaningsih et al. [20], the mean G/L ratio was higher in BC deaths than in live subjects (2.61 vs. 2.11). The G/L ratio considered as a single inflammatory marker was associated with a 2.90-fold increase in BC (per one-point increase) in Polish women, and a 2.35 -fold increase in BC mortality (3rd vs. 1st tertile) in American women [20]. These findings suggest the potential usefulness of the G/L ratio as a new marker in evaluating the association between inflammation and BC. There is some evidence that granulocytes inhibit the cytotoxic effect of lymphocytes and this mechanism might explain the significance of the G/L ratio in cancer-related inflammation [20].

### 4.3. “Pro-Healthy/Neutral-Inflammatory” Profile and Breast Cancer

A higher adherence to the “Pro-healthy/Neutral-inflammatory” profile, characterized by the relatively high consumption frequency of wholemeal cereals/coarse groats, legumes, vegetables, fruits, nuts/seeds and fish, was associated with 62% lower odds of breast cancer occurrence among Polish postmenopausal women. This association remained significant after adjustment for potential confounders only in the continuous model, where the one-point increase in the “Pro-healthy/Neutral-inflammatory” profile score decreased the breast cancer occurrence by 39%. In continuous variable modelling, every one-point change is equal and is more neutral, and allows for the more sensitive detection of differences than a dichotomous model [19]. Consistent with the current results, the “Plant-based” [51], “Vegetables” [52] and “Fruit and Salad” DPs [53] composed of fruits and vegetables only, or “Healthy” DPs, including a Mediterranean diet which also consists of whole grains, nuts and fish, can reduce the risk of BC by 11–56% [38]. However, some studies, including the authors’ previous study [40], did not find a significant association between patterns defined as “Prudent” [39], “Vegetarian” [54] or “Cereals/Milk/Dairy” DPs and breast cancer [55]. A possible explanation of the lack of anti-cancer effect is that these patterns, compared to the “Pro-healthy/Neutral-inflammatory” profile, in addition to fruit and vegetables, also contain foods like breakfast cereals, eggs or cheese which could be considered to be non-healthy when eaten in large amounts [40]. It should be noted that the main components of the pro-healthy patterns like fish and wholemeal cereals, and especially rice and grain-based processed products, contain arsenic, which is recognized as a toxic and a potent carcinogen at high concentrations [56]. Arsenic may also be found in vegetables, legumes, nuts and fruits. In a large cohort study of 1702 Polish women, Marciniak et al. [57] found that chronic low-level exposure to arsenic compounds may lead to a 13-fold increase in breast cancer. The potential mechanism by which arsenic may influence breast cancer development includes estrogen receptor function disruption and estrogen signaling pathway suppression [58].

In the present study, it was also interesting to note that the “Pro-healthy/Neutral-inflammatory” profile was not significantly related to a lower concentration of inflammatory biomarkers. Thus, this profile was neutral to inflammation. This observation is quite surprising because the main components of the “Pro-healthy/Neutral-inflammatory” profile were plant foods and fish. It is well known that bioactive compounds and phytochemicals in plant food have an anti-inflammatory potential [21]. For example, Hermsdorff et al. [24,25] indicated that the consumption of fruits, vegetables, legumes and nuts was associated with decreased serum concentrations of CRP. However, some prospective studies and clinical trials have not found a significant association between higher diet quality linked to lower inflammatory potential and breast cancer risk [33,34,35]. Nevertheless, in addition to the relatively high consumption frequency of pro-healthy foods like wholemeal cereals/coarse groats, legumes, vegetables, fruits, nuts/seeds and fish, the “Pro-healthy/Neutral-inflammatory” profile was characterized by a moderate consumption frequency of foods recognised as non-healthy like sugar/honey/sweets, refined cereals/fine groats, animal fats and red and processed meats. This could cover the anti-inflammatory benefits of bioactive compounds from plant foods and fish and result in a neutral-inflammatory characterisation of the profile [21]. Moreover, the mean blood concentrations of inflammatory markers in Polish women did not exceed the normal level. Furthermore, the anti-cancer result of the “Pro-healthy/Neutral-inflammatory” profile, despite the lack of an inverse association with the inflammatory markers, may indicate the importance of alternative anti-inflammatory pathways in the reducing of the BC occurrence by pro-healthy food consumption [6,21]. A plant-based diet and fish are major sources of bioactive components which may protect against cancer through epigenetic pathways involving DNA repair and cell-cycle regulation [9]. Larouche et al. [9] found that the intake of specific antioxidants, including polyphenols; vitamins A, E, C and β-carotene; some micronutrients like zinc, and selenium; and omega-3 fatty acids, may reduce breast tissue-level inflammation by decreasing oxidative stress and proinflammatory cytokine expression.

### 4.4. Strengths and Limitations

The major strength of the current study is its comprehensive examination of the combined association of dietary and inflammatory factors with breast cancer. To the authors’ best knowledge, no previous study has evaluated the combined effect of blood levels of CRP, IL-6 and G/L ratio and food consumption on breast cancer occurrence. It seems that these markers together may better reflect the inflammatory load and provide a more precise insight into breast cancer etiology compared to each biomarker separately. Secondly, when considering the complex association between hybrid dietary-blood inflammatory profiles and postmenopausal breast cancer, a number of potential confounders have been taken into account in the adjusted model of the logistic regression analysis. This study obtained detailed information on socioeconomic lifestyle factors, including vitamin-mineral supplement use, reproduction and clinical data, which allowed an analysis of confounding effects. Moreover, the study adjusted for BMI and physical activity as two energy-balance covariates, in which BMI was calculated based on the measurement of height, and weight and was not self-declared. Thirdly, since inflammation is linked with cancer in both directions (i.e., it may promote cancer development and growth and could be the host’s response to tumour growth [6]), to avoid the reverse causation, measurements of the inflammatory biomarkers were made just after the primary diagnosis of breast cancer. Chronic inflammation is also a simple marker of other diseases, and circulating levels of CRP or IL-6 are easily influenced by infection and the anti-infectious medication use [17,19]. Therefore, we excluded women with a diagnosis of chronic diseases or infections to prevent false-positive results. This exclusion could limit the impact of those with inflammation due to other causes. Lastly, inflammation may be influenced by many bioactive compounds of functional foods or plant-derived supplements often taken by postmenopausal women to reduce the symptoms of menopause or improve their health condition and beauty [21]. In this study, some women self-reported the use of fibre powder or aloe vera juice, or supplements like lecithin, coenzyme Q10, spirulina, acerola and horsetail. This could impact the outcomes. However, due to the episodic taking of these preparations, it was not necessary to exclude these women. 

Some of this study’s limitations are typical of a case-control design, including biases related to recall and selection [59], which may have led to overestimated results. Since the dietary data were obtained at least one year before the diagnosis, dietary recall can be associated with the respondents’ memory [59]. However, potential recall bias should be small, given the exact face-to-face interview. The second limitation of the study is a relatively small sub-sample size (*n* = 129), which was limited by the restrictive inclusion criteria, including only primary breast cancer cases, lack of other diseases or infections and no medication use. Using a sample smaller than the ideal increases the chance of obtaining overestimated or underestimated results and assuming a false premise as true. However, the sub-sample size calculated in regard to the main objective of the study was greater than the minimum sample size (at least 42 cases and 42 controls) using the WHO sample size calculator (80% test power and 5% significance level) and was sufficient to detect differences between groups [60]. Among the limitations of the study is the non-availability of some food items in the HD-BIPs derived, which were used to calculate DII by Shivappa and colleagues from the world composite database [28,29,30]. However, the missing food parameters include some herbs and spices like ginger, turmeric, thyme, rosemary and saffron, which are consumed in small amounts and infrequently, by the Polish population and their absence may not have had a significant impact on the final results. Moreover, it should be noted that there was no decrease in the predictive capacity of the DII when the number of food parameters had been limited from 45 to 27 into the US validation studies [30]. Other limitations of the study include a single time-point of blood draw and measurement of CRP, Il-6 and leukocyte count, so the results may be influenced by the measurement error and inter-person variation. Multiple biomarker measures would have reduced variability, however, the case-control design of this study did not allow the measurement of markers longitudinally or to assess changes in the time of these levels. However, the available data indicate that a single measurement of CRP and IL-6 concentration, and leukocyte count are useful in population-based studies [14,15,18,19]. It has been observed that the levels of these inflammatory markers from a single blood sample are relatively stable and reflect relatively long-term exposure based on an over 4-year-long follow-up study [12].

## 5. Conclusions

This study revealed that the consumption of highly processed, high in sugar and animal fat foods should be avoided because this unhealthy diet was positively associated with postmenopausal breast cancer occurrence through its pro-inflammatory potential. Instead, the frequent consumption of low-processed plant foods and fish should be recommended since this pro-healthy diet was inversely associated with cancer occurrence even though its anti-inflammatory potential has not been confirmed in this study sample. These findings suggest that monitoring both diet and the blood level of inflammatory markers (including C-reactive protein, Interleukin-6, and the granulocyte-to-lymphocyte ratio) should be considered in a breast cancer reduction strategy, involving screening programs in the early diagnosis of cancer. Furthermore, large prospective studies are needed to explain the mechanisms by which the dietary inflammatory potential leads to elevated inflammatory markers in the aetiology of breast cancer in Polish women, as well as in other populations.

## Figures and Tables

**Table 1:** Frequency of food consumption and the blood concentration of single inflammatory biomarkers (mean (95%CI) or %) by hybrid dietary-blood inflammatory profiles (levels) among postmenopausal women (*n* = 129).

Variable	Hybrid Dietary-Blood Inflammatory Profiles (Levels)					
	**“Pro-healthy/Neutral-inflammatory”**			**“Unhealthy/Pro-inflammatory”**		
	lower (<Me)	higher (≥Me)	*p*-Value	lower (<Me)	higher (≥Me)	*p*-Value
Sample Size (*n*)	64	65		64	65	
**Frequency of food consumption (times/day)**						
Sugar, honey and sweets ^#^	1.82 (1.51; 2.14)	1.61 (1.30; 1.91)	ns	1.14 (0.92; 1.35)	2.29 (1.96; 2.61)	<0.0001
at least once a day	50.0	53.8	ns	34.4	69.2	<0.0001
Wholemeal cereals, coarse groats ^#^	0.65 (0.50; 0.81)	1.41 (1.24; 1.58)	<0.0001	1.08 (0.91; 1.25)	0.99 (0.79; 1.19)	ns
at least once a day	31.3	75.4	<0.0001	56.3	50.8	ns
Refined cereals and fine groats ^#^	1.16 (0.97; 1.34)	0.46 (0.34; 0.57)	<0.0001	0.64 (0.47; 0.80)	0.97 (0.80; 1.14)	0.0015
at least several times/week	78.1	38.5	<0.0001	48.4	67.7	0.0267
Animal fats ^#^	1.11 (0.93; 1.29)	1.26 (1.05; 1.48)	ns	0.73 (0.61; 0.84)	1.64 (1.44; 1.84)	<0.0001
at least once a day	46.9	53.8	ns	28.1	72.3	<0.0001
Fruits ^#^	0.71 (0.63; 0.78)	1.14 (1.03; 1.25)	<0.0001	0.95 (0.85; 1.06)	0.89 (0.79; 1.00)	ns
at least once a day	32.8	89.2	<0.0001	65.6	56.9	ns
Vegetables ^#^	0.93 (0.83; 1.03)	1.38 (1.25; 1.50)	<0.0001	1.17 (1.05; 1.29)	1.15 (1.02; 1.28)	ns
at least once a day	62.5	98.5	<0.0001	84.4	76.9	ns
Nuts and seeds ^#^	0.18 (0.11; 0.25)	0.69 (0.52; 0.86)	<0.0001	0.54 (0.37; 0.71)	0.34 (0.21; 0.46)	0.0254
at least several times/month	43.8	78.5	<0.0001	68.8	53.8	ns
Legumes ^#^	0.09 (0.07; 0.11)	0.26 (0.19; 0.33)	<0.0001	0.16 (0.10; 0.22)	0.19 (0.14; 0.24)	ns
at least several times/month	28.1	72.3	<0.0001	42.2	58.5	ns
Red and processed meats ^#^	1.00 (0.83; 1.16)	1.17 (0.98; 1.36)	ns	0.64 (0.51; 0.77)	1.52 (1.36; 1.67)	<0.0001
at least once a day	45.3	55.4	ns	25.0	75.4	<0.0001
Fish ^#^	0.18 (0.13; 0.24)	0.32 (0.24; 0.40)	0.0326	0.31 (0.23; 0.39)	0.20 (0.14; 0.25)	ns
at least several times/month	50.0	63.1	ns	57.8	55.4	ns
**Blood concentration of inflammatory biomarkers**						
C-reactive protein (mg/L) ^#^	2.26 (1.25; 3.26)	2.87 (1.25; 4.49)	ns	1.23 (0.91; 1.54)	3.89 (2.07; 5.70)	0.0013
≥1.00	51.6	53.8	ns	43.8	61.5	0.0430
Interleukin-6 (pg/mL) ^#^	3.30 (2.33; 4.27)	2.82 (2.34; 3.29)	ns	2.59 (2.26; 2.92)	3.52 (2.51; 4.53)	ns
≥2.30	54.7	47.7	ns	46.9	55.4	ns
Leukocyte count (10^3^ cells/µL) ^#^	6.27 (5.86; 6.67)	6.22 (5.74; 6.69)	ns	5.53 (5.23; 5.83)	6.94 (6.45; 7.43)	<0.0001
≥5.95	57.8	43.1	ns	32.8	67.7	<0.0001
Granulocyte count (10^3^ cells/µL) ^#^	3.77 (3.44; 4.10)	3.70 (3.33; 4.07)	ns	3.15 (2.89; 3.41)	4.31 (3.94; 4.67)	<0.0001
≥3.56	56.3	44.6	ns	26.6	73.8	<0.0001
Neutrophil count (10^3^ cells/µL) ^#^	3.60 (3.27; 3.93)	3.45 (3.09; 3.80)	ns	2.98 (2.72; 3.25)	4.05 (3.69; 4.41)	<0.0001
≥3.31	54.7	46.2	ns	29.7	70.8	<0.0001
Agranulocyte count (10^3^ cells/µL) ^#^	2.49 (2.31; 2.67)	2.58 (2.40; 2.75)	ns	2.38 (2.22; 2.53)	2.69 (2.49; 2.88)	0.0393
≥2.40	56.3	55.4	ns	51.6	60.0	ns
Lymphocyte count (10^3^ cells/µL) ^#^	2.02 (1.85; 2.19)	2.13 (1.98; 2.28)	ns	1.96 (1.81; 2.12)	2.19 (2.02; 2.36)	ns
≥2.00	50.0	50.8	ns	42.2	58.5	ns
Granulocyte-to-Lymphocyte ratio ^#^	2.06 (1.83; 2.29)	1.84 (1.63; 2.05)	ns	1.79 (1.56; 2.03)	2.10 (1.90; 2.31)	0.0094
≥1.75	59.4	40.0	0.0278	35.9	63.1	0.0021

The consumption frequency (categories) was expressed as times/day (values) as follows: “never or almost never” = 0; “once a month or less” = 0.025; “several times a month” = 0.1; “several times a week”= 0.571; “daily” = 1; “several times a day” = 2; Me – median; %—sample percentage; ^#^ mean and 95% confidence interval (95% CI); *p*-value—level of statistical significance verified with Pearson’s chi-squared test (categorical variables) or Kruskal–Wallis test (continuous variables) or Student’s *t*-test (log-transformed biomarkers concentration); *p* < 0.05; ns—statistically insignificant.

**Table 2:** Hybrid dietary-blood inflammatory profiles and the blood concentration of single inflammatory biomarkers in association with postmenopausal breast cancer (mean (95%CI) or %).

Variable	Cancer-Control Sub-Sample	Cancer	Control	*p*-Value
		Sub-Sample	Sub-Sample	
Sample Size	129	47	82	
**“Pro-healthy/Neutral-inflammatory” profile**				
Score (points) ^#^	0.92 (0.62; 1.23)	0.09 (−0.52; 0.70)	1.42 (1.14; 1.70)	0.0001
levels				
lower (<Me)	49.6	64.6	40.7	0.0089
higher (≥Me)	50.4	35.4	59.3	
**“Unhealthy/Pro-inflammatory” profile**				
Score (points) ^#^	5.14 (4.34; 5.95)	6.73 (4.83; 8.63)	4.19 (3.65; 4.73)	0.003
levels				
lower (<Me)	49.6	31.2	60.5	0.0013
higher (≥Me)	50.4	68.8	39.5	
**Blood concentration of inflammatory biomarkers**				
C-reactive protein (mg/L) ^#^	2.58 (1.65; 3.52)	3.85 (1.57; 6.13)	1.80 (1.24; 2.36)	ns
≥1.00	52.7	54	51.9	ns
Interleukin-6 (pg/mL) ^#^	3.06 (2.53; 3.59)	4.06 (2.75; 5.36)	2.46 (2.18; 2.74)	0.006
≥2.30	51.5	61.2	45.7	ns
Leukocyte count (10^3^ cells/µL) ^#^	6.26 (5.95; 6.57)	7.10 (6.48; 7.73)	5.74 (5.46; 6.02)	0.0003
≥5.95	50.4	66	40.7	0.005
Absolute granulocyte count (10^3^ cells/µL) ^#^	3.73 (3.49; 3.97)	4.51 (4.06; 4.95)	3.26 (3.02; 3.49)	<0.0001
≥3.56	50	77.6	33.3	<0.0001
Neutrophil count (10^3^ cells/µL) ^#^	3.54 (3.30; 3.78)	4.39 (3.95; 4.82)	3.02 (2.80; 3.23)	<0.0001
≥3.31	50.4	78	33.3	<0.0001
Absolute agranulocyte count (10^3^ cells/µL) ^#^	2.53 (2.41; 2.66)	2.55 (2.32; 2.79)	2.52 (2.38; 2.67)	ns
≥2.40	55.7	56	55.6	ns
Lymphocyte count (10^3^ cells/µL) ^#^	2.08 (1.96; 2.19)	2.04 (1.84; 2.25)	2.10 (1.96; 2.24)	ns
≥2.00	50.4	48	51.9	ns
Granulocyte-to-Lymphocyte (G/L) ratio ^#^	1.95 (1.80; 2.10)	2.36 (2.12; 2.61)	1.70 (1.52; 1.88)	<0.0001
≥1.75	50	73.5	35.8	<0.0001

Me—median; %—sample percentage; ^#^ mean and 95% confidence interval (95% CI); *p*-value—level of statistical significance verified with Pearson’s chi-squared test (categorical variables) or Kruskal–Wallis test (continuous variables) or Student’s *t*-test (log-transformed biomarkers concentration); *p* < 0.05; ns—statistically insignificant.

## Supplementary Materials

The following are available online at https://www.mdpi.com/2072-6643/12/11/3503/s1, Table S1: Cancer-control sample and its sub-sample characteristics (% or points), Table S2: Cancer-control sub-sample characteristics (% or points), Table S3: Description of selected 10 food groups aggregated: data based on the FFQ-6 questionnaire, Table S4: Description of the socioeconomic status factors, Table S5: Description of the categories of physical activity at work and at leisure time, Table S6: Factor loadings for the frequency of food consumption and the blood concentrations of inflammatory biomarkers in the PCA-derived hybrid dietary-blood inflammatory profiles among postmenopausal women (*n* = 129), Table S7: The cancer-control sub-sample characteristics by dietary-blood inflammatory profiles (%), Table S8: Percentage of breast cancer cases (%), odds ratios (ORs) and 95% confidence interval (95% CI) of postmenopausal breast cancer by adherence to the blood concentration of single inflammatory biomarkers (*n* = 129), Figure S1: Histograms of blood concentrations of interleukin-6 and leukocyte count among control and cancer sub-samples.
