# Supplementary material for: Hybrid Dietary-Blood Inflammatory Profiles and Postmenopausal Breast Cancer: A Case-Control Study
Source: Nutrients. 2020 Nov 14;12(11):3503. doi: 10.3390/nu12113503 (PMC7697398; doi:10.3390/nu12113503)
Supplement: Supplementary file 1 [file nutrients-12-03503-s001.pdf]

Table S1. Cancer-control sample and its sub-sample characteristics (% or points)

Variable	Cancer-Control sample	Cancer-Control sub-sample	*p*-Value	Cancer sample	Cancer sub-sample	*p*-Value	Control sample	Control sub-sample	*p*-Value
Sample Size	420	129		190	47		230	82	
Age (years[#])	59.9 (8.6)	61.9 (8.2)	0.0199	60.9 (9.7)	62.2 (10.4)	ns	59.1 (7.4)	61.7 (6.7)	0.0055
BMI (kg/m^2[#])	27.9 (5.0)	27.9 (5.1)	ns	28.3 (4.8)	28.8 (5.1)	ns	27.6 (5.0)	27.3 (5.1)	ns
Socioeconomic status (SES Index[#])	9.9 (2.1)	9.9 (2.3)	ns	9.3 (2.1)	8.4 (1.8)	0.0074	10.4 (2.0)	10.8 (2.1)	ns
low	41.0	40.8	ns	53.2	69.4	0.0449	30.9	23.5	ns
average	36.7	35.4	ns	33.2	28.6	ns	39.6	39.5	ns
high	22.4	23.8	ns	13.7	2.0	0.0081	29.6	37.0	ns
Overall physical activity									
low	52.9	56.2	ns	67.9	71.4	ns	40.4	46.9	ns
moderate	44.0	41.5	ns	30.5	26.5	ns	55.2	50.6	ns
high	3.1	2.3	ns	1.6	2.0	ns	4.3	2.5	ns
Smoking status (smoker [$])	53.1	46.2	ns	57.9	55.1	ns	49.1	40.7	ns
Abuse of alcohol[@]	4.0	0.0	ns	7.4	0.0	ns	1.3	0.0	ns
Age at menarche (years)									
<12	12.1	7.7	ns	16.3	14.3	ns	8.7	3.7	ns
12-14.9	63.3	70.0	ns	63.2	67.4	ns	63.5	71.6	ns
≥15	24.5	22.3	ns	20.5	18.4	ns	27.8	24.7	ns
Menopausal status									
pre-menopausal	14.8	0.0	0.0001	15.3	0.0	0.0003	14.3	0.0	0.0003
post-menopausal	85.2	100.0	ns	84.7	100.0	0.0003	85.7	100.0	0.0003
Number of full-term pregnancies									
0	12.1	10.8	ns	7.9	4.1	ns	15.7	14.8	ns
1-2	61.7	62.3	ns	61.6	55.1	ns	61.7	66.7	ns
≥3	26.2	26.9	ns	30.5	40.8	ns	22.6	18.5	ns
Oral contraceptive use (ever)	20.2	23.8	ns	17.9	18.4	ns	22.2	27.2	ns
Hormone-replacement therapy use (ever)	16.7	22.3	ns	15.3	20.4	ns	17.8	23.5	ns
Family history of BC[&]	19.3	23.8	ns	24.7	32.7	ns	14.8	18.5	ns
Vitamin/mineral supplements use [α]	38.6	53.8	0.0022	31.1	42.9	ns	44.8	60.5	0.0146
Dietary pattern score (points)[#]									
'Non-Healthy'	3.5 (1.8)	2.9 (1.7)	0.0009	4.1 (1.9)	3.6 (1.7)	ns	3.1 (1.6)	2.5 (1.5)	0.0033
'Prudent'	3.4 (1.2)	3.4 (1.3)	ns	3.3 (1.2)	3.1 (1.5)	ns	3.5 (1.3)	3.5 (1.1)	ns
'Margarine and Sweetened Dairy'	0.1 (1.0)	-0.1 (0.9)	ns	0.2 (1.0)	0.1 (1.0)	ns	0.1 (1.0)	-0.3 (0.8)	0.0012
Molecular subtypes of BC^									
Triple-negative (ER-, PR-, HER2-)	NA	NA	NA	12.1	10.2	ns	NA	NA	NA
ER-, PR-, HER2+ subtype	NA	NA	NA	3.6	0.0	ns	NA	NA	NA
Luminal A (ER+ and or PR+, HER2-)	NA	NA	NA	70.0	73.5	ns	NA	NA	NA
Luminal B (ER+ and or PR+, HER2+)	NA	NA	NA	14.3	16.3	ns	NA	NA	NA

BMI—body mass index; BC—breast cancer; [$]ever-smoker (current and/or former smoker); [@]at least one bottle (0.5 L) of beer or two glasses of wine (300 mL), or two drinks (300 mL), or two glasses of vodka (60 mL) per day [41]; [&]in first- or second-degree relative; [α]self-declared use of vitamin and/or mineral supplements within the last 12 months; [^]data for n=140; ER – oestrogen receptor status of tumour; PR – progesterone receptor status of tumour; HER2 – human epidermal growth factor receptor 2; dietary patterns were derived using the Principal Component Analysis and described previously [40]; NA – not applied; %—sample percentage; [#]mean and standard deviation (SD); *p*-value—level of significance verified with chi^2 test (categorical variables) or Kruskal-Wallis' test (continuous variables); $p < 0.05$; ns – statistically insignificant.

Table S2. Cancer-control sub-sample characteristics (% or points)

Variable	Cancer-Control Sub-Sample	Cancer Sub-Sample	Control Sub-Sample	*p*-Value
Sample Size	129	47	82	
Age (years #)	61.9 (8.2)	62.2 (10.4)	61.7 (6.7)	ns
BMI (kg/m² #)	27.9 (5.1)	28.8 (5.1)	27.3 (5.1)	ns
Socioeconomic status (SES Index #)	9.9 (2.3)	8.4 (1.8)	10.8 (2.1)	0.0001
low	40.8	69.4	23.5	
average	35.4	28.6	39.5	0.0001
high	23.8	2.0	37.0	
Overall physical activity				
low	56.2	71.4	46.9	
moderate	41.5	26.5	50.6	0.0229
high	2.3	2.0	2.5	
Smoking status (smoker $)	46.2	55.1	40.7	ns
Abuse of alcohol @	0.0	0.0	0.0	ns
Alcohol drinking (times/day) # β	0.06 (0.12)	0.02 (0.03)	0.08 (0.14)	0.0001
Age at menarche (years)				
<12	7.7	14.3	3.7	
12–14.9	70.0	67.4	71.6	ns
≥15	22.3	18.4	24.7	
Menopausal status				
pre-menopausal	0.0	0.0	0.0	ns
post-menopausal	100.0	100.0	100.0	
Number of full-term pregnancies				
0	10.8	4.1	14.8	
1–2	62.3	55.1	66.7	0.0084
≥3	26.9	40.8	18.5	
Oral contraceptive use (ever)	23.8	18.4	27.2	ns
Hormone-replacement therapy use (ever)	22.3	20.4	23.5	ns
Family history of BC &	23.8	32.7	18.5	ns
Vitamin/mineral supplements use α	53.8	42.9	60.5	ns
Dietary pattern score # (points)				
'Non-Healthy'	2.9 (1.7)	3.6 (1.7)	2.5 (1.5)	0.0003
'Prudent'	3.4 (1.3)	3.1 (1.5)	3.5 (1.1)	ns
'Margarine and Sweetened Dairy'	−0.1 (0.9)	0.1 (1.0)	−0.3 (0.8)	ns
Food groups# β				
Sugar, honey and sweets	1.72 (1.24)	2.17 (1.46)	1.45 (1.01)	0.0134
Wholemeal cereals and coarse groats	1.04 (0.74)	0.82 (0.73)	1.16 (0.73)	0.0075
Refined cereals and fine groats	0.80 (0.70)	1.03 (0.73)	0.67 (0.66)	0.0051
Animal fats	1.19 (0.80)	1.22 (0.85)	1.17 (0.78)	ns
Fruits	0.92 (0.43)	0.86 (0.43)	0.96 (0.43)	ns
Vegetables	1.16 (0.50)	1.00 (0.43)	1.25 (0.52)	0.0047
Nuts and seeds	0.44 (0.59)	0.35 (0.56)	0.49 (0.61)	ns
Legumes	0.18 (0.23)	0.18 (0.26)	0.17 (0.21)	ns
Red and processed meats	1.08 (0.72)	1.18 (0.73)	1.03 (0.71)	ns
Fish	0.25 (0.28)	0.20 (0.20)	0.28 (0.32)	ns

BMI—body mass index; BC—breast cancer; $ever-smoker (current and/or former smoker); @at least one bottle (0.5 L) of beer or two glasses of wine (300 mL), or two drinks (300 mL), or two glasses of vodka (60 mL) per day [41]; &in first- or second-degree relative; αself-declared use of vitamin and/or mineral supplements within the last 12 months; dietary patterns were derived using the Principal Component Analysis and described previously [40]; βthe consumption frequency was expressed as a times/day after assigning the values for categories of consumption frequency as follows: 'never or almost never'=0; 'once a month or less'=0.025; 'several times a month'=0.1; 'several times a week'=0.571; 'daily'=1; 'several times a day'=2; %—sample percentage; #mean and standard deviation (SD); *p*-value—level of significance verified with chi² test (categorical variables) or Kruskal-Wallis' test (continuous variables); $p < 0.05$; ns – statistically insignificant.

Table S3. Description of selected 10 food groups aggregated: data based on the FFQ-6 questionnaire [40]

No	Food groups	Food groups description
1	Sugar, honey and sweets	Sugar added to beverages, such as tea, coffee, etc.;<br>Honey added to dishes and added to beverages;<br>Chocolates, chocolate sweets and chocolate bars, sugar confectionery (boiled sweets, hard caramels, jellied sweets, fudge, etc.), baked confectionery (biscuits, cream cakes, fruit cakes, sponge cakes, cheesecakes, doughnuts, poppy-seed cakes, muffins, croissants, etc.), ice-creams and custard.
2	Wholemeal cereals and coarse groats	Wholemeal wheat or rye bread, seeded loafs, pumpernickel, wholemeal cracker bread, etc.<br>Buckwheat groats, barley, brown rice, wholemeal pasta, etc.
3	Refined cereals and fine groats	White bread, rye, wheat-rye bread, toast bread, white bread rolls, brioche, bagels, etc.<br>Semolina, milled barley, pasta, white rice, rice flakes, etc.
4	Animal fats	Butter;<br>Lard, pork fat, etc.<br>Cream (single, double, sour, used as an ingredient or added to beverages).
5	Fruits	All kinds of fruits.
6	Vegetables	All kinds of vegetables (potatoes not included).
7	Nuts and seeds	Peanuts, hazelnuts, walnuts, cashews, coconuts, chestnuts, peanut butter, chocolate-nut spread, etc.<br>Pumpkin seeds, sesame seeds, sunflower seeds, wheat germs, wheat bran, etc.
8	Legumes	Fresh and tinned legumes (corn, green peas, green beans, etc.);<br>Dry and processed pulses (beans (fava, butter kidney, broad, French, green), soya, peas, chickpea and processed pulses (baked beans, hummus, other bread spreads)).
9	Red and processed meats	Red meat (pork, beef, veal, etc.)<br>Venison (wild boar, venison, quail, mallard, hare, etc.)<br>Sausages, bacon, reconstituted meat (sausages, meat loaf, hot-dogs, smoked sausages, bacon, etc.)<br>High quality cured meats (ham, poultry and pork-beef good quality cold meats, etc.)<br>Offal products (liver, blood sausage, sweetbread, liver pate, etc.).
10	Fish	Lean fish (pollock, cod, perch, hake, carp to 1 kg, tuna, panga, trout, etc.)<br>Oily fish (salmon, sardines, herring, mackerel, eel, large carp, etc.).

Table S4. Description of the socioeconomic status factors [40]

Socioeconomic factors	Categories	Scoring
place of residence	village	1
	town <20,000	2
	town 20,000-100,000 inhabitants	3
	city >100,000 inhabitants	4
educational level	primary	1
	secondary	2
	higher	3
economic situation (self-declared)	below average	1
	average	2
	above average	3
situation of household (self-declared)	we live poorly—I don't have enough resources even for basic needs (food/clothing/housing fees)	1
	we live very thriftily—I have enough resources only for basic needs (food/clothing/housing fees)	2
	we live thriftily—so I have enough resources for everything	3
	we live well—I have enough resources for everything, but I don't put off savings	4
	we live very well—I have enough resources for everything and I put off savings	5

Scoring - values assigned to the response categories.

Table S5. Description of the categories of physical activity at work and at leisure time [40]

Physical activity	Categories	Description
at work	low	more than 70% of working time spent sedentary or retired
	moderate	50% of working time spent sedentary and 50% of working time spent in an active manner
	high	70% of working time spent in an active manner or physical work related to great exertion
at leisure time	low	sedentary for most of the time, watching TV, reading books, walking 1–2 h/week
	moderate	walking, bike riding, gymnastics, gardening, light physical activity performed 2–3 h/week
	high	bike riding, jogging, gardening, sport activities involving physical exertion performed more than 3 h weekly

Table S6. Factor loadings for the frequency of food consumption and blood concentrations of inflammatory biomarkers in PCA-derived profiles among postmenopausal women (n=129)

Food groups/ Inflammatory biomarkers	Hybrid dietary-blood inflammatory profiles	
	**'Pro-healthy/Neutral-inflammatory'**	**'Unhealthy/Pro-inflammatory'**
Wholemeal cereals and coarse groats	**0.68**	-0.03
Refined cereals and fine groats	**-0.62**	**0.30**
Legumes	**0.60**	0.04
Vegetables	**0.59**	0.01
Fruits	**0.59**	-0.04
Nuts and seeds	**0.58**	-0.20
Fish	**0.34**	-0.27
Red and processed meats	0.03	**0.70**
Animal fats	0.01	**0.66**
C-reactive protein	-0.08	**0.65**
Sugar, honey and sweets	-0.07	**0.53**
Granulocyte-to-lymphocyte (G/L) ratio	-0.11	**0.52**
Interleukin-6	-0.22	**0.32**
Share in explaining the variance (%)	22	13

Bolded values are marked for the main components of PCA-derived profiles with absolute factor loadings ≥|0.30|.

Table S7. Cancer-control sub-sample characteristics by PCA-derived profiles (% or mean (SD))

Variable	Hybrid dietary-blood inflammatory profiles (levels)					
	'Pro-healthy/Neutral-inflammatory'			'Unhealthy/Pro-inflammatory'		
	lower (<Me)	higher (≥Me)	***p*-Value**	lower (<Me)	higher (≥Me)	***p*-Value**
Sample size (n)	64	65		64	65	
Age (years[#])	63.1 (9.1)	60.4 (7.3)	0.0426	61.6 (8.7)	61.9 (8.1)	ns
BMI (kg/m^{2}[#])	28.6 (4.7)	27.1 (5.4)	0.0423	27.4 (5.2)	28.3 (5.1)	ns
Socioeconomic status (SES Index[#])	9.4 (2.3)	10.4 (2.2)	0.0046	10.6 (2.3)	9.2 (2.1)	0.0002
low	46.9	33.8		23.4	56.9	
average	39.1	32.3	0.0299	39.1	32.3	<0.0001
high	14.1	33.8		37.5	10.8	
Overall physical activity						
low	59.4	52.3		48.4	63.1	
moderate	37.5	46.2	ns	46.9	36.9	ns
high	3.1	1.5		4.7	0.0	
Smoking status (smoker [$])	43.8	47.7	ns	39.1	52.3	ns
Abuse of alcohol [@]	0.0	0.0	ns	0.0	0.0	ns
Alcohol drinking (times/day) [# β]	0.07 (0.15)	0.06 (0.11)	ns	0.07 (0.13)	0.05 (0.13)	ns
Age at menarche (years)						
<12	9.4	6.2		4.7	10.8	
12-14.9	68.8	70.8	ns	68.8	70.8	ns
≥15	21.9	23.1		26.6	18.5	
Number of full-term pregnancies						
0	10.9	10.8		15.6	6.2	
1-2	54.7	70.8	ns	62.5	63.1	ns
≥3	34.4	18.5		21.9	30.8	
Oral contraceptive use (ever)	25.0	23.1	ns	23.4	24.6	ns
Hormone-replacement therapy use (ever)	17.2	27.7	ns	31.3	13.8	0.0179
Family history of BC[&]	20.3	27.7	ns	23.4	24.6	ns
Vitamin/mineral supplements use [α]	45.3	61.5	ns	54.7	52.3	ns
Dietary pattern score [#] (points)						
'Non-Healthy'	3.4 (1.5)	2.5 (1.8)	0.0007	1.8 (1.2)	4.0 (1.4)	<0.0001
'Prudent'	2.5 (0.7)	4.2 (1.1)	<0.0001	3.3 (1.2)	3.4 (1.3)	ns
'Margarine and Sweetened Dairy'	0.1 (0.9)	-0.3 (0.8)	0.0141	0.1 (0.7)	-0.3 (0.9)	0.0041

BMI—body mass index; BC—breast cancer; [$]ever-smoker (current and/or former smoker); [@]at least one bottle (0.5 L) of beer or two glasses of wine (300 mL), or two drinks (300 mL), or two glasses of vodka (60 mL) per day [41]; [&]in first- or second-degree relative; [α]self-declared use of vitamin and/or mineral supplements within the last 12 months; dietary patterns were derived using the Principal Component Analysis and described previously [40]; [β]the consumption frequency was expressed as a times/day after assigning the values for categories of consumption frequency as follows: 'never or almost never'=0; 'once a month or less'=0.025; 'several times a month'=0.1; 'several times a week'=0.571; 'daily'=1; 'several times a day'=2; Me – median values; %—sample percentage; [#]mean and standard deviation (SD); *p*-value—level of significance verified with chi^{2} test (categorical variables) or Kruskal-Wallis' test (continuous variables); $p < 0.05$; ns – statistically insignificant.

Table S8. Percentage of breast cancer cases (%), odds ratios (ORs) and 95% confidence interval (95% CI) of postmenopausal breast cancer by adherence to the blood concentration of single inflammatory biomarkers (n = 129)

Variable	Levels	Sample Size	Percentage (%)	*p*-Value	Breast Cancer					
					Unadjusted Model 1		Model 2		Model 3	
					ORs	95% CI	ORs	95% CI	ORs	95% CI
**C-reactive protein (mg/L)**	lower (ref.)	87	36		1.00 (referent)		1.00 (referent)		1.00 (referent)	
	higher (≥1.00)	42	43	0.4479	1.09	0.53; 2.23	0.69	0.28; 1.66	0.75	0.28; 1.99
	1-unit increase				1.10	0.99; 1.21	1.09	0.99; 1.20	1.07	0.97; 1.18
**Interleukin-6 (pg/mL)**	lower (ref.)	89	28		1.00 (referent)		1.00 (referent)		1.00 (referent)	
	higher (≥2.30)	40	60	0.0005	1.88	0.91; 3.89	1.71	0.69; 4.23	1.96	0.71; 5.43
	1-unit increase				1.41**	1.10; 1.79	1.42**	1.09; 1.85	1.50**	1.11; 2.02
**Leukocyte count (10^3 cells/μL)**	lower (ref.)	86	28		1.00 (referent)		1.00 (referent)		1.00 (referent)	
	higher (≥5.95)	43	58	0.0010	2.82**	1.35; 5.92	2.29	0.92; 5.69	2.01	0.73; 5.50
	1-unit increase				1.63***	1.27; 2.11	1.63***	1.22; 2.17	1.65**	1.18; 2.30
**Absolute granulocyte count (10^3 cells/μL)**	lower (ref.)	86	25		1.00 (referent)		1.00 (referent)		1.00 (referent)	
	higher (≥3.56)	43	63	<0.0001	6.91***	3.03; 15.73	5.62***	2.20; 14.38	4.88**	1.73; 13.76
	1-unit increase				2.17***	1.53; 3.07	2.22***	1.51; 3.27	2.33***	1.49; 3.62
**Neutrophil count (10^3 cells/μL)**	lower (ref.)	86	24		1.00 (referent)		1.00 (referent)		1.00 (referent)	
	higher (≥3.31)	43	67	<0.0001	7.09***	3.12; 16.12	6.16***	2.36; 16.04	6.21***	2.16; 17.84
	1-unit increase				2.51***	1.72; 3.67	2.63***	1.71; 4.04	2.78***	1.72; 4.50
**Absolute agranulocyte count (10^3 cells/μL)**	lower (ref.)	88	37		1.00 (referent)		1.00 (referent)		1.00 (referent)	
	higher (≥2.40)	41	41	0.6003	1.02	0.49; 2.10	0.78	0.33; 1.88	0.53	0.20; 1.42
	1-unit increase				1.06	0.65; 1.74	0.90	0.49; 1.68	0.80	0.40; 1.58
**Lymphocyte count (10^3 cells/μL)**	lower (ref.)	86	38		1.00 (referent)		1.00 (referent)		1.00 (referent)	
	higher (≥2.00)	43	40	0.8219	0.86	0.42; 1.75	0.54	0.22; 1.33	0.39	0.14; 1.08
	1-unit increase				0.88	0.51; 1.53	0.70	0.35; 1.43	0.59	0.26; 1.32
**Granulocyte-to-lymphocyte (G/L) ratio**	lower (ref.)	65	20		1.00 (referent)		1.00 (referent)		1.00 (referent)	
	higher (≥1.75)	64	55	<0.0001	4.97***	2.26; 10.92	6.13***	2.39; 15.68	5.93***	2.09; 16.79
	1-point increase				2.52***	1.55; 4.07	2.76***	1.56; 4.88	2.90***	1.53; 5.49

ref. – referent, the reference categories were the control sample and the lower level concentration of single biomarkers; Model 2—age (years), BMI (kg/m^2), socioeconomic status (low, average, high), overall physical activity (low, moderate, high), smoking status (non-smoker, smoker), alcohol drinking (times/day), age at menarche (<12, 12–14.9, ≥15 years), number of full-term pregnancies (0, 1–2, ≥3), oral contraceptive use (no, yes), hormone-replacement therapy use (no, yes), family history of breast cancer in first- or second-degree relative (no, I don't know, yes), vitamin/mineral supplements use (no, yes) and molecular subtypes of breast cancer (triple-negative, ER-, PR-, HER2+ subtype, luminal A, luminal B) adjusted model; Model 3—model was adjusted for the same variables included in model 2 plus PCA-driven DPs score (fully-adjusted model); *p*-value—the level of significance verified with Wald's test; * $p < 0.05$, ** $p < 0.01$, *** $p < 0.001$.

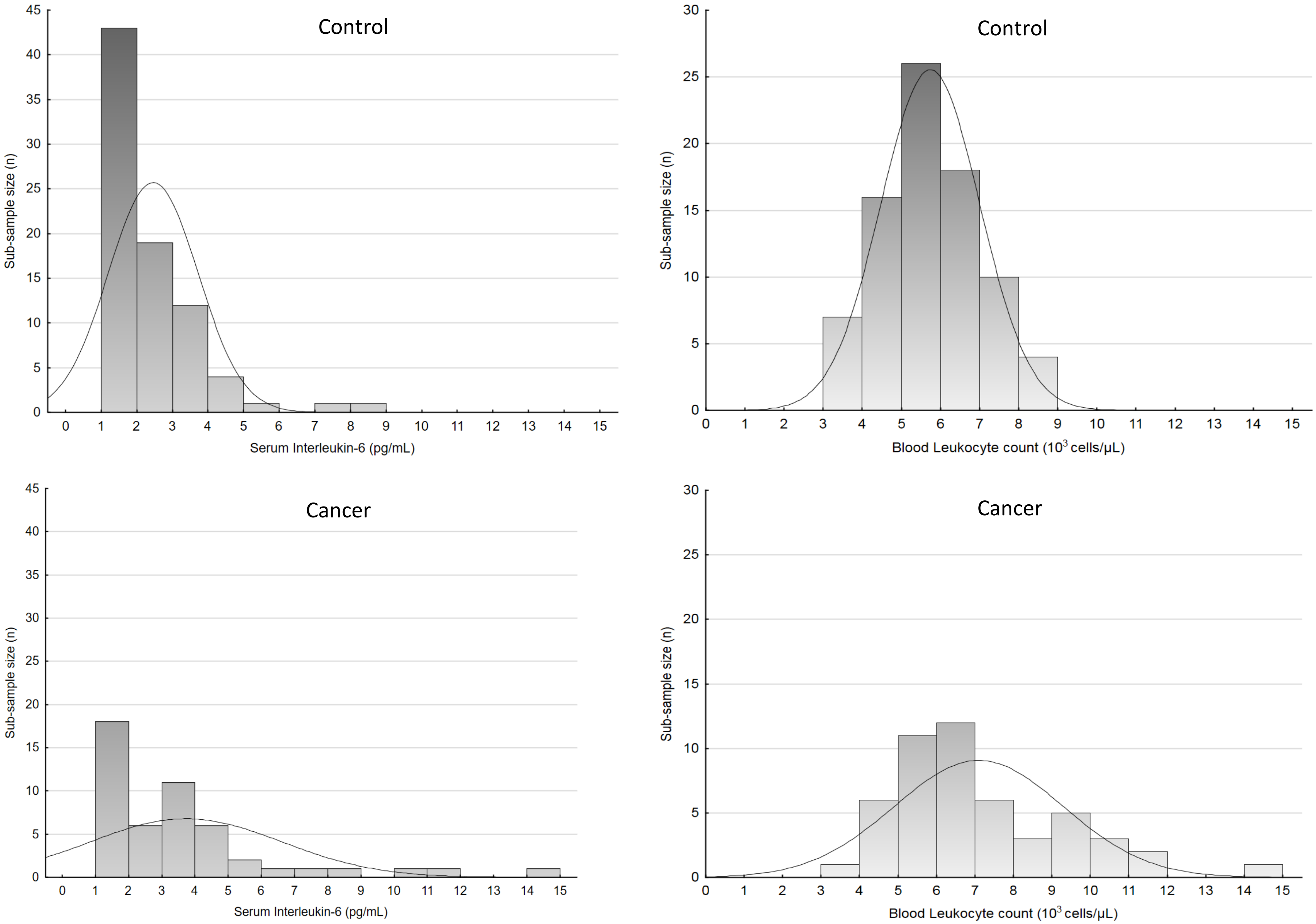

Figure S1. Histograms of blood concentrations of interleukin-6 and leukocyte count among control and cancer sub-samples
